# Spatial distribution pattern of dominant tree species in different disturbance plots in the Changbai Mountain

**DOI:** 10.1038/s41598-022-18621-x

**Published:** 2022-08-19

**Authors:** Mengtao Zhang, Jin Wang, Xingang Kang

**Affiliations:** 1 College of Forestry, Shanxi Agriculture University, Shanxi, 030801 People’s Republic of China; 2 Aerospace Information Research Institute, Chinese Academy of Sciences, Beijing, 100101 People’s Republic of China; 3 Key Laboratory for Silviculture and Conservation Ministry of Education, Beijing Forestry University, Beijing, 100083 People’s Republic of China

**Keywords:** Ecology, Forest ecology

## Abstract

The effects of disturbance on spatial patterns and species interactions provide critical information on community structure and species coexistence. Two standard plots of 1-ha were set, one of them was a sample plot with retrograde succession after disturbance, and the other one was undisturbed. Spatial indices and Spatial patterns statistics were used to analyze the spatial pattern and inter-specific correlation of main tree species in two plots. Our results showed that the diameter distributions of different species have reverse J-shape, unimodal and bimodal distribution in the disturbed plot, while bell-shaped curves were observed in the undisturbed plot. The distributions of tree species were mainly showed clustered patterns in almost all scales through univariate pair correlation function. Some similar results of the classification of Wiegand scheme of species association consistent with the consequences of the bivariate pair correlation. The mark variograms showed positive autocorrelation at a coarse scale. The current study may aid in efforts of forest management planning and ecosystem services. Meanwhile, different research methods of spatial distribution also help to improve the accuracy of spatial distribution and the interspecific association of tree species.

## Introduction

The spatial patterns of trees and their interactions provide critical information on community structure, species coexistence and significantly determine reproduction, growth, mortality, dispersal, resource use, gap creation, and understory development^1,2^. Especially for the natural forest, the complex spatial structure directly affects the seed spreading and the rejuvenation process of seedlings, resulting in the change of stand space. Therefore, it is imperative to evaluate the unpredictable potential processes through the current forest structure^3^, starting point for the change of natural forest succession. Disturbance is the driving force of forest succession, the degree of disturbance determines the direction of stand development and structure, including regeneration, species composition, community dynamics, and light mechanism of the forest^4–6^.

Coniferous and broad-leaved mixed forest is the main forest type in Changbai Mountain area, and this kind of forest usually shows high species richness and unique species composition^7^. It is important to understand the biological characteristics and potential ecological processes of forests in this area^8^. In recent decades, due to various disturbances (natural disasters and unreasonable logging), a large number of natural secondary forests appeared in this area, while coniferous and broad-leaved mixed forest resources with primitive characteristics were very scarce. This phenomenon had a significant negative impact on the productivity and function of the forest^9,10^. For the deepening of people's understanding of the protection of forest resources in this area, a large number of studies had been carried out, such as the status and change of ecosystem service^11^, modeling stand growth dynamics^12^, the mechanism of forest regeneration^13^, biodiversity^14^, the developing status or behavior of plant communities^15^, etc. However, there are relatively few studies on the changes in stand structure, especially under different succession stages and disturbance^16,17^.

The existing studies mainly focused on the distribution of tree size, the degree of size heterogeneity, the spatial correlation of trees in different forest layers, and various methods, including distance-related spatial structure function and distance-independent spatial structure–function. For example, Hao et al. investigated spatial patterns and associations of main tree species among different tree height layers using *O*-ring statistics, which was based on a 25-ha broad-leaved Korean pine (*Pinus koraiensis*) mixed forest plot of Changbai Mountain of northeastern China^18^. The results indicated that the spatial pattern and associations of main tree species were closely related to tree height class and environmental heterogeneity. Zhao et al. used variance to mean ratio, cluster index, and Cassie index to examine the horizontal distribution patterns of saplings in the spruce-fir coniferous and broad-leaved mixed forest of Changbai Mountain and their results showed that the saplings were mainly distributed in clusters, and the degree of clustering decreased with the increase of height^19^. Li et al. reported the priority of felling trees based on the distance-independent spatial indexes (uniform angle index; mingling index and dominance index) and believed that using these indicators can reduce the subjectivity of the selection process and improved the speed and accuracy of the choice of trees to be harvested in uneven-aged mixed forests^20^. The above-mentioned studies only considered the spatial distribution or interspecific correlation of tree species and did not give reasonable explanations for the results produced by different methods.

In this study, two standard plots of 1-ha were set, one of them was a sample plot with retrograde succession after disturbance, and the other was an undisturbed sample plot. Spatial pattern function, pair correlation function, Weigand classification scheme and marker variation were used to analyze the spatial pattern and interspecific correlation of main tree species in two plots. We assumed that: (i) the spatial patterns of species and their interspecific correlation are related to scale, and the proportion of species composition; and (ii) using different spatial indices and functions to analyze spatial patterns may produce different results. Based on the above-mentioned assumptions, we tried to solve the following research questions:

1. What is the species distribution pattern of dominant tree species in different disturbance plots?
2. Is the result of the spatial index consistent with the spatial function?
3. Is there any spatial correlation between tree diameters, and if so, is the performance of the diameter correlation consistent with the spatial scale correlation?

## Results

### Stand Structure

Table 1 shows the results of structural characteristics for species in the entire plots. *Betula platyphylla* (*Bp*) ranked as the most dominant in disturbed plot, with the highest values of a number of individuals (664) and basal area (11.321 m^2^/ ha), as shown in Table 1. However, only 214 trees existed in undisturbed plot, in which basal area was 3.385 m^2^/ha. *Pinus koraiensis* (*Pk*) had the largest DBH in both disturbed and undisturbed plots, which was 56.2 cm and 55.5 cm, respectively. *Populus davidiana* (*Pd*) and *Betula costata* (*Bc*) only existed in the disturbed plot, and their basal area were 2.136 and 0.706 m^2^/ha, which ranked third and fourth, respectively. *Acer mono* (*Am*) and *Tilia amurensis* (*Ta*) ranked third and fourth in undisturbed plot, with density 168 and 121, and basal area 1.592 and 1.784 m^2^/ha, respectively. The range of Gini index was from 0.36 to 0.47 and 0.61 to 0.75, respectively.

**Table 1:** Structural characteristics for species in the entire plots.

	Species	Mean DBH (cm)	Max DBH (cm)	Standard deviation	Basal area (m^2^/ha)	Density (n/ha)	Density proportion of species	Gini index
Disturbed plot	*Betula platyphylla* Suk. (Betulaceae)	13.9	29.3	4.89	11.321	664.0	0.49	0.37
	*Pinus koraiensis Sieb. et Zucc.* (Pinaceae)	8.70	56.2	4.35	2.986	401.0	0.30	0.47
	*Populus davidiana* Dode (Salicaceae)	11.50	31.9	4.83	2.136	175.0	0.13	0.43
	*Betula costata* Trautv. (Betulaceae)	8.32	19.0	2.78	0.706	117.0	0.09	0.36
Undisturbed plot	*Pinus koraiensis Sieb. et Zucc.* (Pinaceae)	17.74	55.5	10.06	23.215	711	0.58	0.70
	*Betula platyphylla* Suk. (Betulaceae)	12.78	34.3	6.23	3.385	214	0.18	0.66
	*Acer mono* Maxim. (Aceraceae)	10.02	37.5	4.51	1.592	168	0.14	0.61
	*Tilia amurensis* Rupr. (Tiliaceae)	11.65	42.0	7.23	1.784	121	0.10	0.75

**Figure 1:**
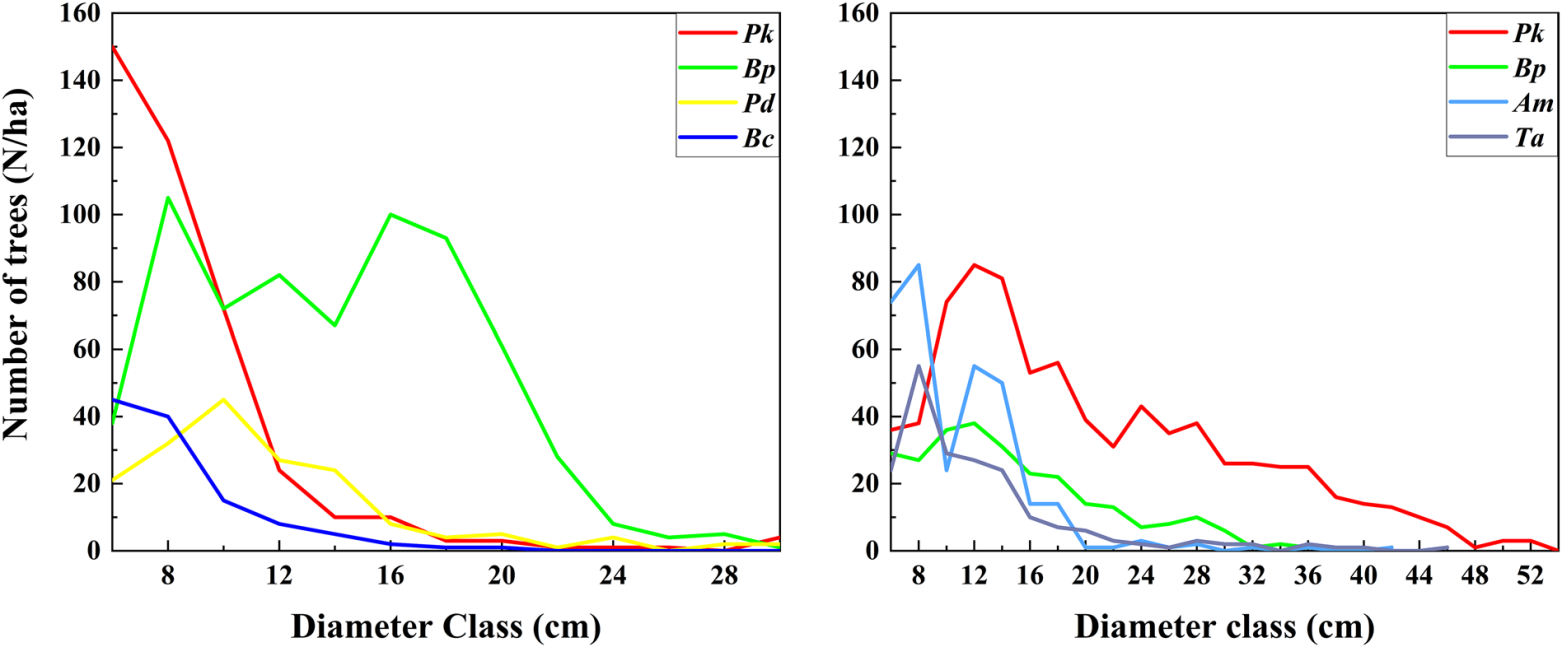
Diameter distributions of four dominant species trees in both disturbed (left) and undisturbed (right) plots. Red color represents *Pinus koraiensis Sieb. et Zucc.* (*Pk*), green color represents *Betula platyphylla* Suk. (*Bp*), yellow color represents *Populus davidiana* Dode (*Pd*), bule color represents *Betula costata* Trautv. (*Bc*), cyan color represents *Acer mono* Maxim. (*Am*), and purple color represents *Tilia amurensis* Rupr. (*Ta*). The color in the following figure is the same meaning.

The diameter distributions of different species are shown in Fig. 1. In disturbed plot, *Pk* and *Bc* had reverse J-shape curves. *Pd* had a unimodal diameter distribution, while *Bp* showed an almost bimodal distribution. In an undisturbed plot, the diameter distributions of four tree species showed bell-shaped curves, and number of trees first increased and then decreased with increasing size.

### Species uniform angle indices and spatial distribution patterns

**Figure 2:**
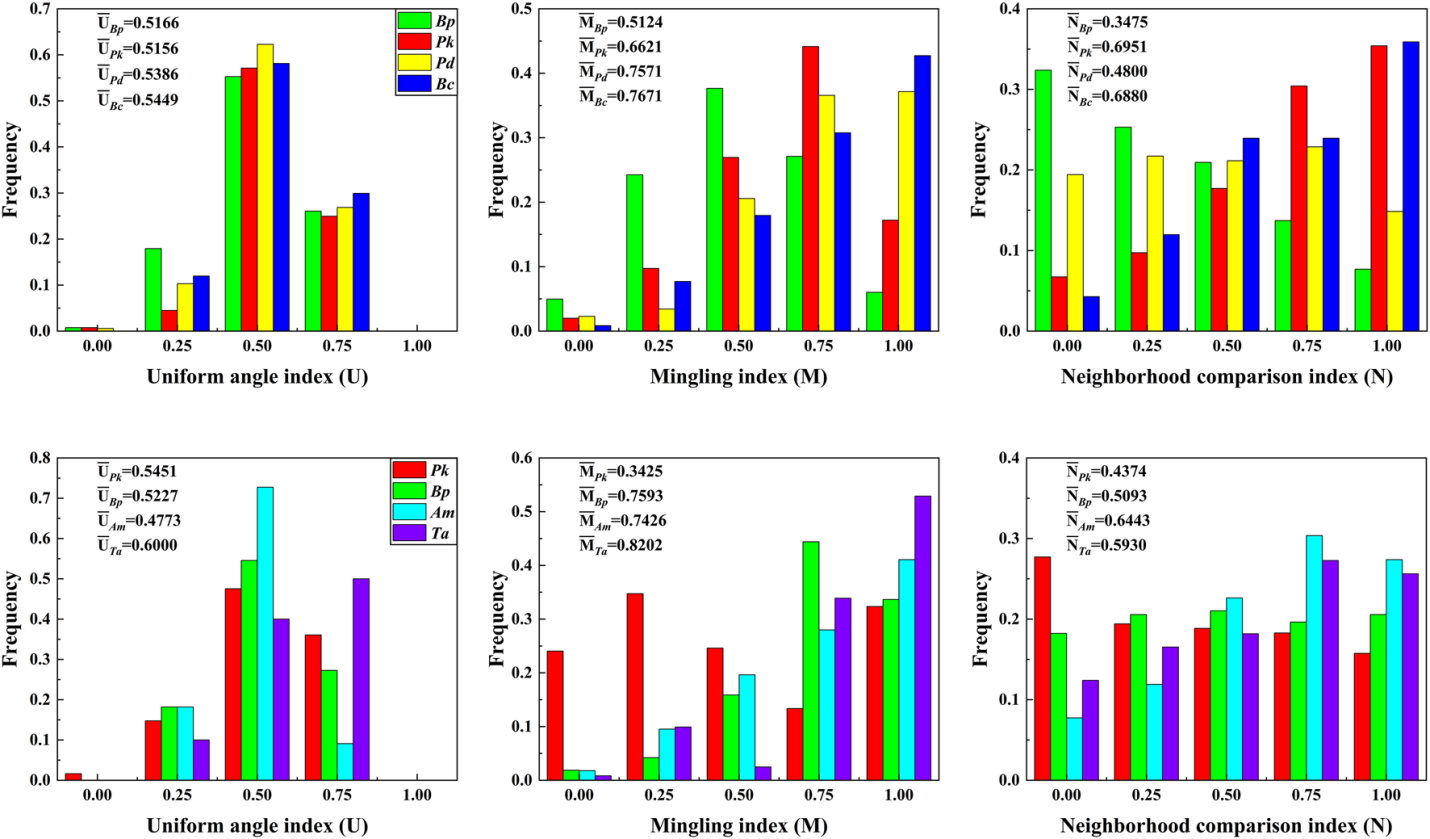
Frequency distribution of the structural indices for dominant species in the disturbed (above) and undisturbed (below) plots.

**Figure 3:**
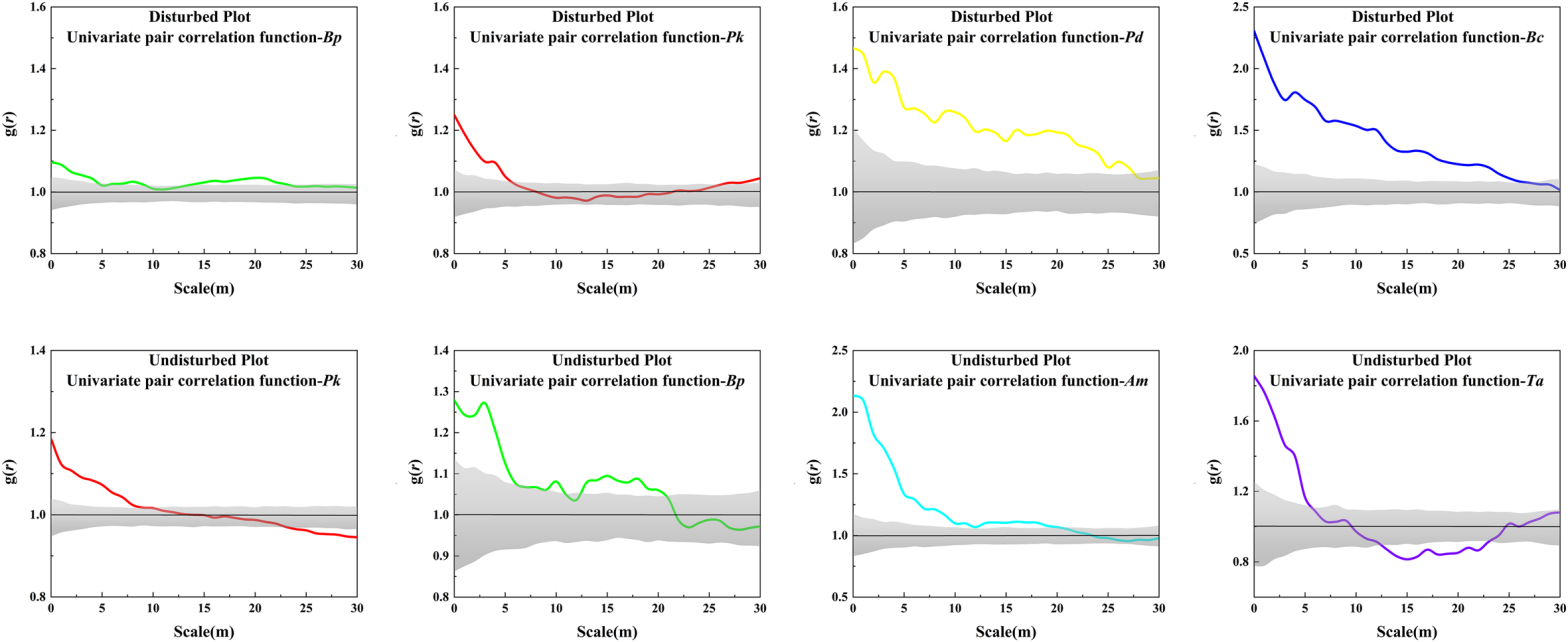
Spatial patterns of the four dominant tree species in both disturbed (above) and undisturbed (below) plots. The Y axes represents the g(*r*) values, which is an index of spatial pattern; the X axes represents the spatial distance scales. Black solid lines correspond to expected values for a random distribution. Different colored solid lines show actual g(*r*) values, gray shaded area represents 95% confidence intervals generated from 499 Monte Carlo simulations under the null hypothesis of complete spatial randomness.

Frequency distributions of the spatial structural indices are shown in Fig. 2. The uniform angle indices of all species in both entire plots mainly distributed from 0.5166 to 0.5449 and 0.4773 to 0.6000, indicating that the distributions of tree species were mainly random. In the disturbed plot, weak uniform distribution occurred except *Bc*. However, only *Pk* showed similar weak homogeneity in the undisturbed plot. When the value of the uniform angle indices was equal to 0.75, there were significant differences in two plots. The frequency of four species exhibited essentially the same in the disturbed plot, while *Pk*, *Bp* and *Ta* exhibited higher inhomogeneity than *Am*. The univariate spatial distributions of all trees in both plots exhibited in Fig. 3. Trees of *Bp* showed clustered distribution between 0–9 and 14–23 m scales in the disturbed plot. *Pk* trees were observed aggregated at short (0–5 m) scales. Some species, such as *Pd* and *Bc* in the disturbed plot, showed clustered distributions in most of the scales. In the undisturbed plot, trees of *Pk* and *Ta* showed clustered distribution at 0–8 m and 0–5 m scales, while regular trends at 24–30 m and 13–23 m scales, respectively. *Bp* and *Am* trees exhibited aggregated distributions at scales between in 0–7 m, 13–20 m and 0–2 0 m. And then, these trends became random distributions at large interval scales, respectively.

### Species mingling and neighborhood comparison index

The average mingling indexes varied from 0.5124 to 0.7671 and 0.3425 to 0.8202, respectively. In the disturbed plot, weak, middle and strong mingling could be observed in species *Bp*. while the frequencies of the mingling index in species *Pk*, *Pd* and *Bc* showed an increasing trend in the disturbed plot. In the undisturbed plot, the species *Bp*, *Am* and *Ta* showed a high level of mixing, especially the mingling index of *Ta* was more distributed in 0.75 and 1.00 values. The species *Pk* was distributed in all values of the degree of mingling indexes, but the degree of weak and middle mingling was higher than the degree of strong and extremely strong. The frequency of neighborhood comparison index distributed ranges from 0.3475 to 0.6951 and 0.4374 to 0.6443 in both disturbed and undisturbed plots (Fig. 2).

In the disturbed plot, the neighborhood comparison index of *Bp* was mainly distributed in the values of 0.00, 0.25, and 0.5, and these values explained that the diameters with more than 80% target trees were larger than those of neighbor trees, which was in the absolute dominant position. Frequency distributions of *Pk* and *Bc* mainly focused on the values of 0.5, 0.75 and 1.00, which explained that these two species existed smaller than neighbors. The species *Pd* distributed evenly among all the values, and the average neighborhood comparison index was closed to 0.5 (0.4800). *Pk* was the chief species in the undisturbed plot, but the degree of dominance (frequency in the value 0.00) was inconspicuous. The average neighborhood comparison index of *Bp* trees was closed to 0.5 (0.5093), which was in the moderate position. The frequencies of an index in *Am* and *Ta* was mainly distributed in the values of 0.75 and 1.00, which explained that these two species existed smaller trees than neighbors. However, these trends changed slowly in this plot (Fig. 2).

### Species associations and classification scheme

In the disturbed plot, *Bp* was positive correlated to *Pk* at scales ≤ 5 m and 19–21 m, tendencies towards segregation emerged at another scale. However, *Bp* and *Pd* trees showed significant aggregation at 8–30 m scales. On the contrary, *Bc* trees were negatively associated with *Bp* and *Pk* trees at larger scales. The trends of positive associations between *Pk*-*Pd* and *Pd*-*Bc* species pairs increased with scales, but species pairs of *Pd*-*Bc* showed negative association at scales ≤ 12 m. In the undisturbed plot, segregation trend of species pair of *Pk* and *Bp* was observed at ≤ 13 m scales, and when the scales increased, independent and positive associations emerged. In almost all scales, *Pk* and *Am* trees exhibited spatial independence. Trees of *Ta* were also negatively associated with *Pk* at scales 0–9 m. Species of *Bp* was conspicuous aggregated with *Am* and *Ta* at 0–16 m and 0–9 m scales, but this aggregate trend in species of *Bp*-*Ta* changed independent and segregation with the scales (17–30 m) accumulated. *Am* trees showed segregation versus *Ta* trees at short scales (0–3 m) and a tendency toward segregation at larger scales (27–30 m). More details can be seen in the supplementary Table S2.

**Figure 4:**
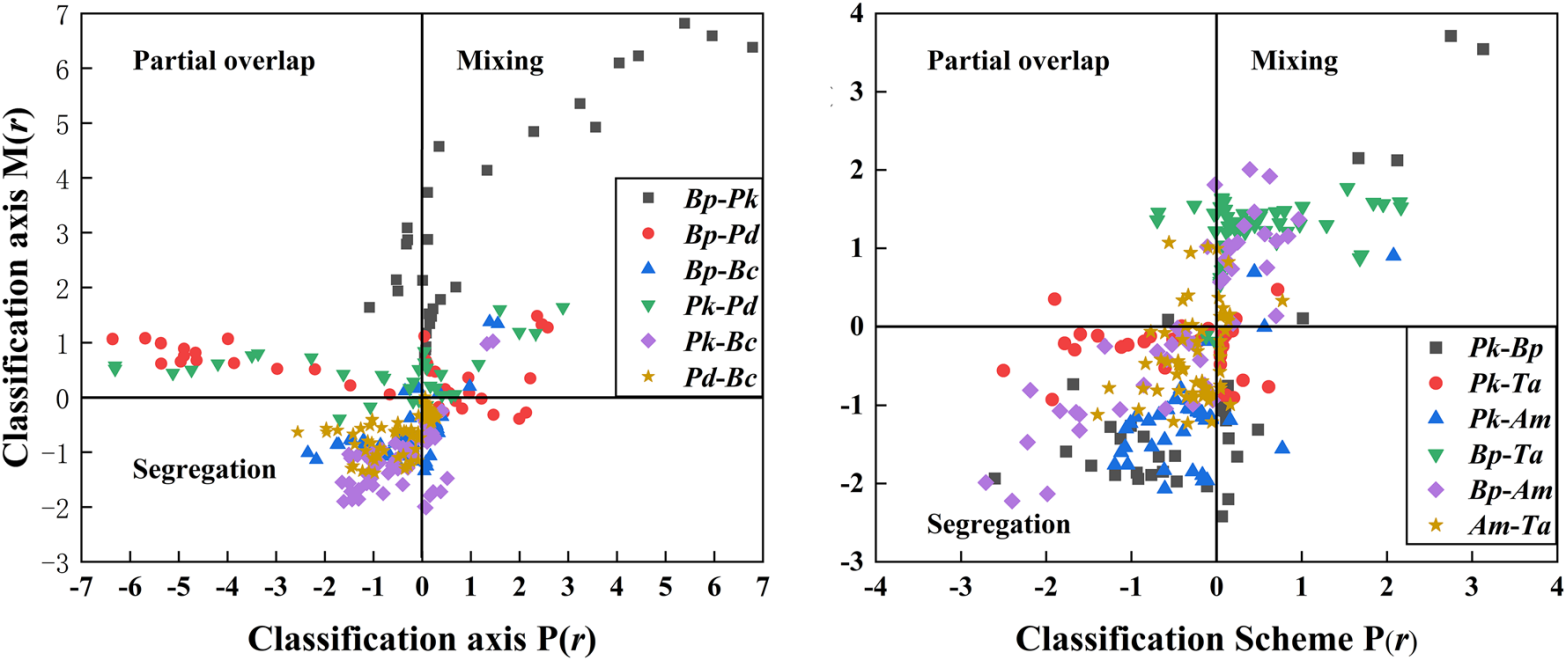
Species association classification using the Wiegand scheme in both disturbed (left) and undisturbed (right) plots.

The results of species association classification using the Wiegand scheme were basically consistent with the consequences of the bivariate pair correlation (Fig. 4). In addition, there were some partial overlaps among species pairs of *Bp*-*Pk*, *Bp*-*Pd* and *Pk*-*Pd* in the disturbed plot, which could be an indication of site homogeneity. However, in the undisturbed plot, partial overlaps were almost not existed among species pairs, which could be proof of the site heterogeneity.

### Species size diversity

**Figure 5:**
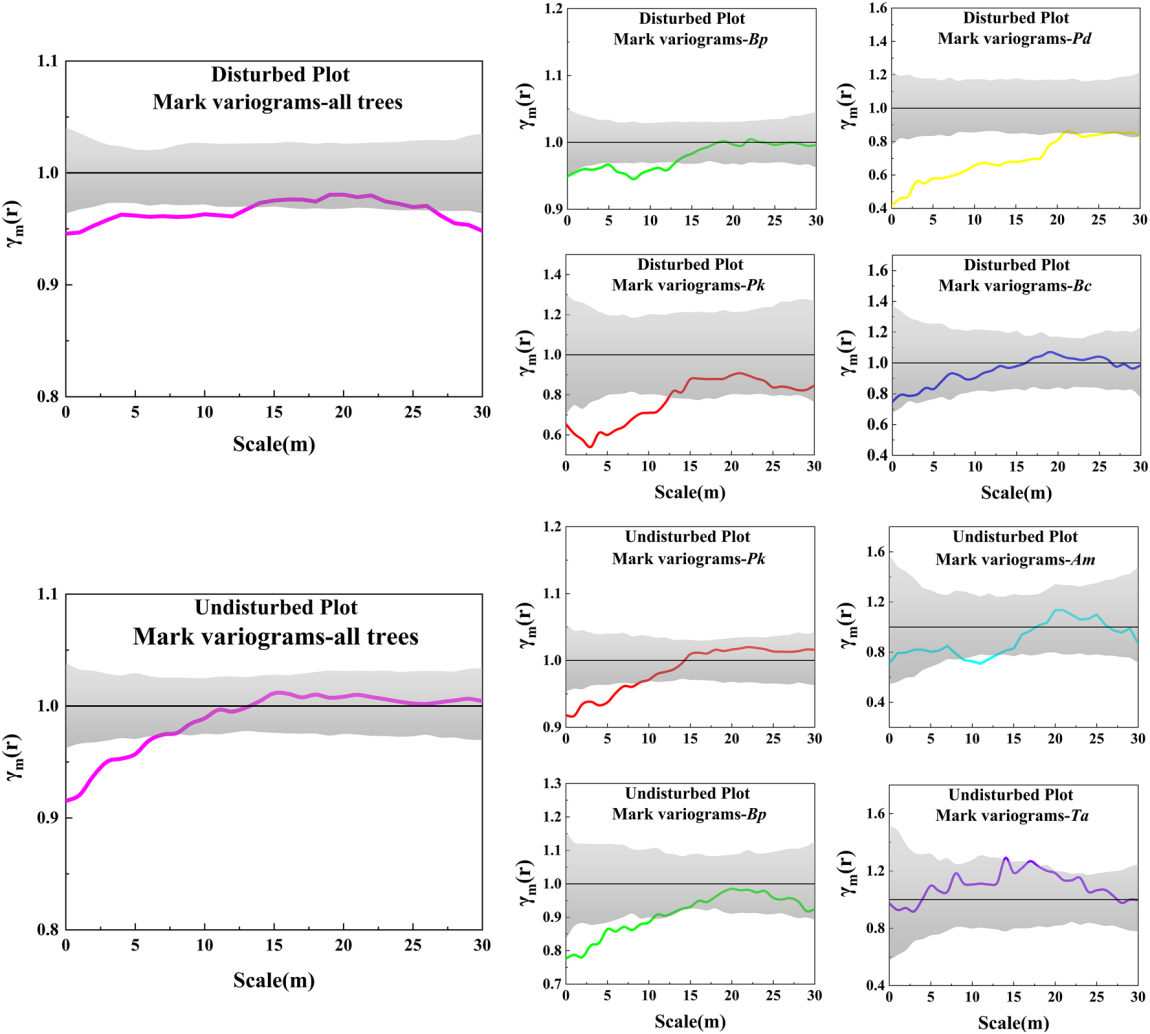
Mark variograms for dominant species in the disturbed and undisturbed plot. The Y axes represents the γ_m_(*r*) values, which is a measure of the similarity of the marks (here tree DBH) depending on the distance between points; the X axes represents the spatial distance scales. Black solid lines *γ*_m_ = 1 correspond to the values for the case with independent marks. Different colored solid lines show actual γ_m_(*r*) values, where pink color represents all trees and gray shaded area indicates upper and lower of the limit 99% confidence envelopes.

Mark variogram showed positive correlations among all tree species at scales ≤ 12 m, 27–30 m and ≤ 6 m in two plots (Fig. 5). Consistent with the results of spatial distribution patterns (Fig. 2), and combined with the consequence of neighborhood comparison index (Fig. 1), mark variogram in *Bp* trees indicated that aggregation between large diameter *Bp* trees and other small-diameter trees occurred in the disturbed plot. Similarly, the mark variograms of *Pk* and *Pd* trees showed clumped distributions among the disturbed plot and the competition pressure between neighbors and references were also greater. The mark variogram of *Bc* trees showed no spatial correlation of tree diameter between co-dominant neighbors and references at any scale. Trends of mark variogram in trees of *Pk* and *Bp* were similar to the result of all trees. According to Fig. 1, mark variogram in *Pk* trees also indicated an aggregation between large diameter *Pk* trees and other small-diameter trees. In contrast to *Pk* trees, *Am* and *Ta* trees showed spatial no correlation at most scales. Only at scales of 14 m and 17 m, species of *Ta* showed a negative autocorrelation.

## Discussion

After suffering from a serious disturbance, the natural forest can be divided into secondary, deforested and primary forest, according to the level of degradation^21^. The forest type of the undisturbed plot is broadleaved Korean pine forest. Korean pine forest, also called primary forest, which shows limited effects of human disturbance, has maintained its basic primitive forest form and relatively high stand stability^22,23^. The results of Table 1 and Fig. 1 are consistent with the spatial characteristics. These findings were attributed to the selective logging which took place 36 years ago, and most broad-leaved pioneer trees, such as *Bp* and *Pd*, regenerated under the canopy gaps^24^. As the dominant tree species, *Pk* kept the diameter distribution rule of the natural forest, but in the disturbed plots, the number of trees decreased. Univariate pair correlation function *g*(*r*) for most standing trees showed clustered patterns in almost all scales, except *Pk* trees. The species *Pk* in the disturbed plot was observed that aggregate pattern at fine scales, which could be attributable to the limited seed dispersal ability of the tree species, include the character of tree seeds (e.g., the larger-sized pine cones of coniferous tree species), or the spread ability of seeds was obstructed by the complex shape of breaches^25–27^. Environmental heterogeneity might have been contributed to the aggregated distributions at large scales^28,29^. The same result was observed in the undisturbed plot, in which the *Pk* trees showed a clumped distribution at scales 0–8 m. However, Trees of *Bp* and *Am* exhibited aggregation predominantly at two-thirds (< 20 m) scales. Besides environmental heterogeneity, this phenomenon might also be ascribed to logging, and several canopy gaps were created. Some intolerant tree species, such as birch and maple, got more light resources, grew good under the gaps, and showed agminated trends at coarse scales^30,31^. Forest spatial patterns are closely related to the distance scale^32,33^. However, there was some discrepancy between the uniform angle index and the pair correlation function. Shaaban et al. considered that the uniform angle index could not distinguish aggregation patterns in most cases^27^. Río et al. doubt about the measurement advantage of the angle measurement between neighborhoods in the distance-independent method^34^. The spatial distribution of trees may fit one pattern at one interval and another at a different interval^35^. This may suggest that the uniform angle index lacks the analysis and prediction of large-scale spatial patterns and can only analyze the spatial pattern between adjacent trees.

The value of the species mingling index not only reflects the average mixing state of a stand, but also expresses the diversity of tree species^36^. For the species mingling index, trees of *Bp* showed a low average value (0.5124) in the disturbed plot, while a high average value (0.7593) in the undisturbed plot. This result suggested that: (1) higher diversity in the undisturbed plot than the disturbed plot; (2) with the advance of progressive succession, the status of pioneer species was gradually reduced; (3) the proportion of *Bp* trees was decreased with an increasing degree of mixing. Trees of *Pk*, on the contrary, exhibited the opposite trend from *Bp* in the mingling index, which showed a high average value (0.6621) in the disturbed plot, and a low average value (0.3425) in the undisturbed plot. Graz discovered that the mingle degree of tree species in stand decreased with the increase of tree species composition proportion, and suggested that the mingling index was sensitive to the proportion of the species^37^. Our results are consistent with the findings of the Graz’s study. Similarly, trees of *Pd* and *Bc* in the disturbed plot, and trees of *Am* and *Ta* in the undisturbed plot, kept a high mingling index, because of their low species density proportion (Table.1).

Through bivariate pair correlation function and the Wiegand scheme with species association, we found that the species of *Pd* was positively associated with *Bp* and *Pk* at large scales (≥ 8 m and ≥ 14 m) in the disturbed plot. Since trees of *Bp* and *Pk* were ranked the first and second-largest individuals, and as the above mentioned *Pk* trees, meanwhile, were mainly small trees, these phenomena were not surprising. These findings are consistent with the previous work^38^. In the classification of species association using the Wiegand scheme, we found similar results, such as *Bp* trees was mixing with *Pk*, partial overlap with *Pd*, and segregation with Bc. The species pairs of *Pk* and *Bp* were also positively associated at specific scales, which suggested limited seed dispersal of the coniferous species, and *Pk* could thrive under *Pd* or *Bp* canopies^39^. Trees of *Bc* was negatively associated with the other three species at coarse scale intervals, which illustrated that canopies of some tree species provide unsuitable habitats for the recruitment of *Bc*, presumably through attenuation of incoming radiation^31^. We conclude that different species have different habitat requirements for persistence and recruitment^40^. In addition, the number of *Bc* trees was lower in the disturbed plot, indicating that the negative correlation was credible. In the undisturbed plot, trees of *Pk* were negatively associated with *Bp* and *Ta* at 0–13 m and 0–9 m scales, while no relevancies was observed between the species pairs of *Am* and *Pk*, and *Am* and *Ta* at the most scales. *Pk* was the dominant species in this plot, and the limited resources available could not meet the demand for all trees. Therefore, “self-thinning” likely triggered among the co-dominant or intermediate trees, so as to produce spatial segregate and independent^41^. The results of classification of species association showed the same. However, trees of *Bp* were positively associated with *Am* and *Ta* at some scales, which attributed to these tree species forming such situations in the early stage of succession^8^. According to the Wiegand scheme classification, trees of *Bp* was mixed with *Ta* and *Am*, but some segregation trends also exhibited between *Bp* and *Am*.

Shaaban analyzed the size differentiation between the reference and the neighbor trees using the differentiation index. However, this index is sometimes easy to confuse, that is, the partial information of the degree of differentiation cannot accurately determine whether the reference tree is surrounded by thicker adjacent trees^27^. Therefore, this study used the neighborhood comparison index which could more accurately quantify the degree of size differentiation among trees^42^. Combined with the mark variograms, the size relationship between the reference and the neighbor trees was further explained accurately. According to the neighborhood comparison index, *Bp* was the dominant species in the disturbed plot, and the nearly 80% proportion of more than two neighbor trees whose DBH was less than the reference tree. Meanwhile, combined with the results of the mark variograms, it was known that the diameters of *Bp* trees were larger than that of their adjacent trees with positive autocorrelation at fine scales. In this plot, the number of *Pk* trees was ranked only second to *Bp* trees. However, *Pk* was at a disadvantage in niche, which also proved that most of *Pk* trees grown under the canopy of *Bp* trees, which could provide good shade conditions for the growth of *Pk* trees, making *Pk* become the dominant tree species in the later stage of succession^43^. The neighborhood comparison index of *Pd* trees was close to 0.5, but the mark variograms showed obvious positive autocorrelation at a coarse scale, indicating that there were both big and small diameter sized positive autocorrelation neighbors with *Pd* trees. The small number of *Bc* trees showed the spatial no correlation of tree diameter.

Suzuki et al. specified that negative autocorrelation was uncommon in natural forests in which trees are distributed in a spatially random or clustered way, which is the instance in our research. In the undisturbed plots, the neighborhood comparison index of *Pk* and *Bp* showed that they were in the dominant and moderate positions, respectively^44^. According to the mark variograms, the young recruited trees of *Pk* were mainly aggregated with big diameter *Pk* trees on a particular scale (< 10 m), which also confirmed the seed dispersal mechanism of *Pk*^18^. Although *Bp* tended to be moderate, according to the results of the uniform angle index and bivariate pair correlation function *g*(*r*), the diversity of tree species gathered around the reference was higher than that of *Pk* trees, indicating that broad-leaved tree species had a strong tillering ability in the process of growth.

## Conclusions

In this study, we used the spatial index and spatial pattern statistics to explore the spatial pattern and interspecific correlation of main tree species in the mixed forest under different degrees of disturbance and the spatial correlation of DBH of tree species based on Mark variograms. The findings of the current study may aid in efforts of forest management planning and ecosystem services. Tree species that showed positive correlations, such as species of *Pk*, can grow better under the crown of broad-leaved trees (*Bp* and *Ta*), which provided a reference basis for tree species after-culture of artificial aids to natural regeneration in disturbed plots. Meanwhile, different research methods of spatial distribution also help to improve the accuracy of spatial distribution and the interspecific association of tree species. However, due to the limited experimental conditions, we did not include saplings of DBH < 5 cm in our analysis. Therefore, there was a lack of results on spatial distribution between adult trees and saplings. Besides, the assembly of long-term monitoring data should be combined to analyze the influence of interference mechanism on the spatio-temporal dynamic change of tree species patterns in future research.

## Materials and methods

### Study area

**Figure 6:**
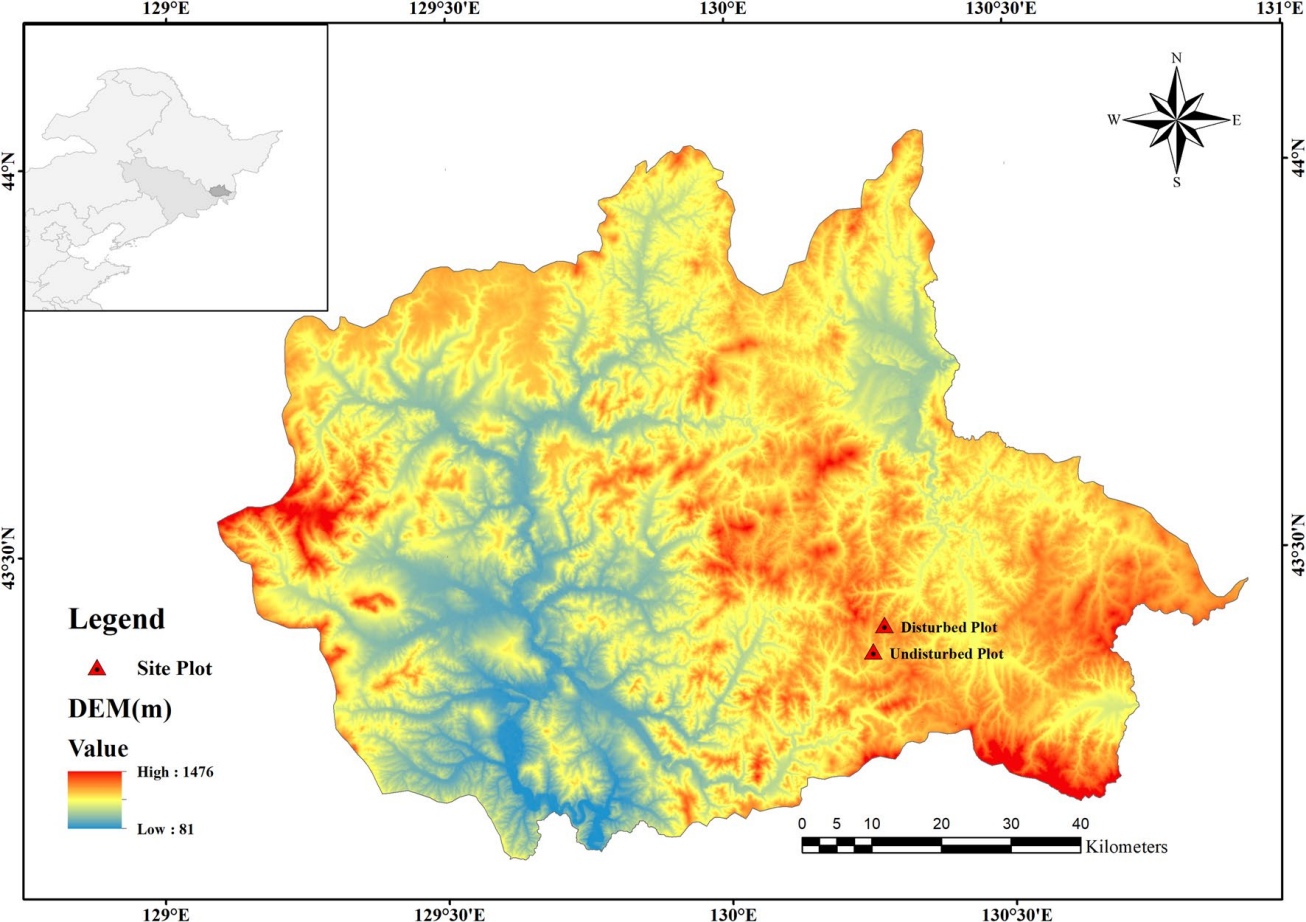
Location map of disturbed and undisturbed plots in Wangqing County, Northeast China.

The study area is located in Wangqing County, Yanbian Autonomous Prefecture, located at Jilin province, in northeastern China (43° 06ʹ–44° 03ʹ N, 129° 51ʹ–130° 10ʹ E) (Fig. 6). This region belongs to the monsoon climate. The annual average temperature ranges from 3.7 to 4.2 °C, and the annual precipitation ranges from 580 to 700 mm. The frost-free period is 110 to 140 days. The number of days with annual snow cover ranges from 70 to 100 days, with a depth of 20–30 cm. The annual sunshine is 2700 h.

Before the nineteenth century, there was little human disturbance in the Changbai Mountains' forests, but during the twentieth century, human disturbance became more frequent and severe, and the area of primary forest decreased by 70.4 percent from 1950 to 1986, unreasonable logging and overgrazing^45,46^. Because Korean pine is a dominant tree species of primary forest in northeastern China. These forests are the most productive, such as the wood quality of Korean pine is very good, and the seed can be bought at a higher price. Therefore, these forests are threatened by Human activities^31,47^. After suffering from a serious disturbance, the natural forest can be divided into secondary and primary forest, according to the level of degradation. In this study, we chose these two forest types as disturbed and undisturbed sample plots.

### Data collection and analysis

We established the plot in an unevenly aged polar-birch secondary natural forest in 2012. The trees of this plot were formed by succession after logging (1986). The next year (2013), the other plot was established in the natural broad-leaved Korean pine forest that suffered from little disturbance because the plot was in the demonstration area in the local area. The acreage of both plots was 100 m × 100 m. Each plot was divided into regular grid 25 subplots of 20 × 20 m, and the grid intersections were marked with metal pipes. The coordinates, diameter at breast height (DBH), height of the standing free trees with > 5 cm DBH, which included juveniles and mature trees were measured in each subplot. The individuals were identified by their scientific species names.

The importance value (IV) is a comprehensive quantitative indicator used to characterize the status and role of each species in a community. In this research, IV was composed of relative density, relative frequency and relative dominance, which were equal to one-third of the sum of the above-mentioned three indexes. Detailed descriptions of the IVs can be found in Zhang et al.^26^. Finally, according to the ranking of important values, the top four tree species were selected as the main research tree species in both disturbed & undisturbed plots. The dominant tree species are *Betula platyphylla* Suk. (Betulaceae), *Pinus koraiensis* Sieb. et Zucc. (Pinaceae), *Populus davidiana* Dode (Salicaceae), *Betula costata* Trautv (Betulaceae), *Acer mono* Maxim (Aceraceae) and *Tilia amurensis* Rupr (Tiliaceae), more detailed description could be seen in the supplementary Table S1.

Both spatial indices in short distances (e.g., uniform angle index, mingling index, and neighborhood comparison index) and spatial functions (univariate and bivariate pair correlation function, mark variogram, and the Weigand scheme) were used to assess the spatial distribution of trees, respectively.

### Spatial indices

Uniform angle index (*U*_*i*_) describes the degree of regularity of the four trees' spatial distribution nearest to a target tree *i*. The concept is based on the classification of the angles *α*_*j*_ (*j* from 1 to n) between the immediate neighbors of the n trees with reference to a target tree. Uniform angle index is defined as the ratio of all the reference angles *α*_*j*_ smaller than the standard angle *α*_*0*_ to the total number of *α*_*j*_^48^. The *U*_*i*_ formula is as follows:

1 $$\begin{gathered} U_{i} = \frac{1}{4}\sum\limits_{j = 1}^{4} {z_{ij} } \hfill \\ z_{ij} = \left\{ \begin{gathered} 0, \, if\alpha_{j} < \alpha_{0} \hfill \\ 1,{\text{ otherwise }} \hfill \\ \end{gathered} \right.{\text{ and }}0 \le U_{i} \le 1 \hfill \\ \end{gathered}$$

The degree of isolation of tree species can be expressed as the mingling index (*M*_*i*_). The mingling index describes the degree of spatial isolation of tree species in mixed forests and is defined as the proportion of the four nearest neighboring trees of another species^48^. It is expressed by the formula:

2 $$\begin{gathered} M_{i} = \frac{1}{4}\sum\limits_{j = 1}^{4} {z_{ij} } \hfill \\ z_{ij} = \left\{ \begin{gathered} 0,{\text{ neighbor }}j{\text{ belongs to the same species as the reference tree }}i \hfill \\ 1,{\text{ otherwise}} \hfill \\ \end{gathered} \right. \hfill \\ {\text{ and }}0 \le M_{i} \le 1. \hfill \\ \end{gathered}$$

The neighborhood comparison index (*N*_*i*_) can be expressed as the competitive pressure of forest building group species and is defined as the size differentiation between neighboring trees and is calculated based on the ratio between thinner and thicker DBH of four nearest neighboring trees. The formula of *N*_*i*_ is as follows:

3 $$\begin{gathered} N_{i} = \frac{1}{4}\sum\limits_{j = 1}^{4} {k_{ij} } \hfill \\ k_{ij} = \left\{ \begin{gathered} 0,{\text{ if neighbor }}j{\text{ is smaller than the reference tree }}i \hfill \\ 1,{\text{ otherwise}} \hfill \\ \end{gathered} \right. \hfill \\ {\text{ and }}0 \le N_{i} \le 1 \hfill \\ \end{gathered}$$

### Spatial patterns statistics

Comparing with the Ripley’s *K* function, the pair-correlation function *g*(*r*) is closely related to the second-order product density and can effectively prevent cumulative effects. The *g*-function was calculated as follows:

4 $$g(r) = \frac{dK(r)}{{dr}}/2\pi r,{\text{ for }}r > 0$$

The univariate pair correlation function is the expected density of points within a given distance *r* of an arbitrary point, divided by the intensity of the pattern. The bivariate pair correlation function is the ratio of the observed mean density of points of pattern 2 at distance *r* of an arbitrary point of pattern 1 to the expected mean density of pattern 2. When the spatial pattern of the trees is complete spatial randomness (CSR), the value of *g*(*r*) = 1 means that the trees are randomly distributed and independent association in a certain scale, the value of *g*(*r*) > 1 means aggregation and positive association, and *g*(*r*) < 1 means regularity and negative association over the entire plot^49,50^.

All associations of the tree species may show high variability; therefore, a two-dimensional classification space based on the two summary statistics *K*_12_(*r*) and *D*_12_(*r*) was used^51^. *K*_12_(*r*) was the bivariate Ripley *K* function and *D*_12_(*r*) was the nearest-neighbor distribution. The expectations of the two summary statistics under both the CSR and independence null models yield ${\mathrm{D}}_{12}\left(\mathrm{r}\right)=1-exp(-{\uplambda }^{2}\pi {\mathrm{r}}^{2})$ and ${\mathrm{K}}_{12}\left(\mathrm{r}\right)=\pi {\mathrm{r}}^{2}$. Both *K*_12_(*r*) and *D*_12_(*r*) were implemented to construct the scheme's two axes. The two axes P(r) and M(r) are defined as follows:

5 $$\begin{gathered} P(r) = \frac{{(D_{12} (r) - E[D_{12} (r)]}}{{SD[D_{12} (r)]}} \hfill \\ M(r) = \frac{{(K_{12} (r) - E[K_{12} (r)]}}{{SD[K_{12} (r)]}} \hfill \\ \end{gathered}$$

where ${D}_{12}\left(r\right)$ and ${K}_{12}\left(r\right)$ are estimated from observed data and the operators *E*[.] and *SD*[.] indicate the expectation and standard deviation of summary statistics at the neighborhood *r* under independence, respectively. The theoretical value of the two summary statistics *K*_12_(*r*) and *D*_12_(*r*) under the null model are subtracted from those observed to set the case of null association *P*(*r*) = *M*(*r*) = 0^52^.

The statistics *M*(*r*) and *P*(*r*) evaluate two fundamental aspects of bivariate point patterns. If the proportion of the nearest neighbors within the range *r* is less than expected, *P*(*r*) value will be negative and vice versa. The distribution of *P*(*r*) and *M*(*r*) under the null model can be approximated by the standard normal distribution, thus the box delimited by values of [–2.33, 2.33] (which correspond to a *p*-value of 0.025 for two summary statistics individually) approximates the area where the null hypothesis cannot be rejected, and a given species departs more strongly from independence the farther away it is located from the box. Several fundamental types of spatial association patterns are possible for each neighborhood *r*^53,54^:

Type 0: no departures: neither *K*_12_(*r*) nor *D*_12_(*r*) show significant departure from the null model.

Type I: Segregation: Species pairs located in the lower-left quadrant show segregation because there are fewer individuals of species *j* within neighborhoods of radius *r* around individuals of species *i* than expected under the null model (*M*(*r*) < 0 and *P*(*r*) < 0).

Type II: Partial overlap: Species pairs located in the upper-left quadrants show partial overlap because individuals of species *j* occur more often within neighborhoods of radius *r* around individuals of species *i*, but a notable proportion of individuals of species *i* have fewer neighbors of species *j* than expected under the null model (*M*(*r*) > 0 and *P*(*r*) < 0).

Type III: Mixing: Species pairs located in the upper-right quadrant show a high degree of spatial association (mixing) because here individuals of species *j* occur more often within neighborhoods of radius r around individuals of species *i*, and individuals of species *i* have more neighbors of species *j*, than expected under the null model (*M*(*r*) > 0 and *P*(*r*) > 0).

Type IV: This association type is predicted to occur rarely when trees of species *i* are highly aggregated and few trees of species *j* overlap the cluster of species *i* (*M*(*r*) < 0 and *P*(*r*) > 0).

The spatial correlation of tree diameter was analyzed using a mark variogram. The mark variogram, *γ*_m_(*r*) is a measure of the similarity of the marks (here tree DBH) depending on the distance between points and provides two critical characteristics: range from correlation and the strength of interaction. The definition of mark variogram can be described in following equation:

6 $$\gamma_{m} = 1/2E(m(x)) - (x + r)^{2} /\sigma_{m}^{2}$$

where, *x* and *x* + *r* denote the locations of two arbitrary points. When the distribution of trees is independent of the tree diameters, *γ*_m_(*r*) takes the value of 1. In the presence of segregation, correlation is negative and *γ*_m_(*r*) > 1. In contrast, a positive correlation indicates that the pairs of trees tend to have similar marks and results in *γ*_m_(*r*) < 1.

### General tests of hypotheses for spatial patterns statistics

The complete spatial randomness (CSR) null hypothesis was used in the spatial distribution of each tree species^36^. We postulated that taller tree height classes suppress the recruitment and growth of lower layers for the bivariate statistic, but lower layers do not affect higher trees^55^. Therefore, we fixed the upper layer tree height class locations and randomized the locations of the lower tree height class using a Poisson cluster null model. The null hypothesis for spatial correlation of tree diameters was complete spatial independence of the tree diameter distribution. This hypothesis was tested using the random labelling test^56^.

For all the analyses, we used 499 randomizations in the null model of Monte Carlo simulations to provide 99% confidence intervals^57^. To avoid edge effects, translation correction was used in the analyses. The spatial scale of distribution patterns (univariate) was selected in 0–25 m range, which is 1/4 of the minimum plot dimension^58^. The interspecific association was 0–30 m range, because of interaction between individuals can affect a limited scale and a larger scale has no biological significance^1^. To standardize, we chose 0–30 m range as the pattern scales. The *Winkelmass* 1.0 and *Programita* (2018) were used to compute the spatial indices and spatial patterns statistics^51,59^.

## Supplementary Information

Supplementary Information.

## Data Availability

The data used to support the findings of this study are available from the corresponding author upon request.
