# Supplementary material for: Spatial distribution pattern of dominant tree species in different disturbance plots in the Changbai Mountain
Source: Sci Rep. 2022 Aug 19;12:14161. doi: 10.1038/s41598-022-18621-x (PMC9391346; doi:10.1038/s41598-022-18621-x)
Supplement: Supplementary file 1 — Supplementary Information. [file 41598_2022_18621_MOESM1_ESM.docx]

Supplementary Table 1. Rank of the dominant tree species in disturbed & undisturbed plots according to the important values.

|  | Species | Number of trees (n/ha) | Relative density | Relative frequency | Relative dominance | Importance value |
| --- | --- | --- | --- | --- | --- | --- |
| Disturbed plot | *Betula platyphylla* Suk. (Betulaceae) | 664 | 0.40 | 100.00 | 54.97 | 51.79 |
|  | *Pinus koraiensis Sieb. et Zucc.* (Pinaceae) | 401 | 0.24 | 100.00 | 14.49 | 38.24 |
|  | *Populus davidiana* Dode (Salicaceae) | 175 | 0.11 | 84.00 | 10.37 | 31.49 |
|  | *Betula costata* Trautv. (Betulaceae) | 117 | 0.07 | 84.00 | 4.62 | 29.56 |
|  | *Tilia amurensis* Rupr. (Tiliaceae) | 71 | 0.04 | 68.00 | 3.51 | 23.85 |
|  | *Larix gmelinii* Kuzeneva (Pinaceae) | 23 | 0.01 | 56.00 | 2.50 | 19.51 |
|  | *Syringa reticulata* H. Hara var. (Oleaceae) | 53 | 0.03 | 56.00 | 0.77 | 18.94 |
|  | *Picea jezoensis* Carr. (Pinaceae) | 19 | 0.01 | 52.00 | 0.66 | 17.56 |
|  | *Abies nephrolepis* Trautv. Maxim (Pinaceae) | 93 | 0.06 | 44.00 | 6.17 | 16.74 |
|  | *Acer mono* Maxim. (Aceraceae) | 33 | 0.02 | 48.00 | 1.77 | 16.60 |
|  | *Acer tegmentosum* Maxim. (Aceraceae) | 5 | 0.00 | 16.00 | 0.11 | 5.37 |
|  | *Malus baccata* Linn. ( Rosaceae) | 2 | 0.00 | 8.00 | 0.03 | 2.68 |
|  | *Fraxinus mandschurica* Rupr. (Oleaceae) | 3 | 0.00 | 4.00 | 0.07 | 1.36 |
|  | *Ulmus pumila* Linn. (Ulmaceae) | 2 | 0.00 | 4.00 | 0.06 | 1.35 |
| Undisturbed plot | *Pinus koraiensis Sieb. et Zucc.* (Pinaceae) | 717 | 0.58 | 100.00 | 75.87 | 58.82 |
|  | *Betula platyphylla* Suk. (Betulaceae) | 214 | 0.17 | 100.00 | 11.17 | 37.11 |
|  | *Acer mono* Maxim. (Aceraceae) | 168 | 0.14 | 100.00 | 5.25 | 35.13 |
|  | *Tilia amurensis* Rupr. (Tiliaceae) | 121 | 0.10 | 96.00 | 6.93 | 34.34 |
|  | *Phellodendron amurense* Rupr. ( Rutaceae) | 4 | 0.00 | 12.00 | 0.25 | 4.08 |
|  | *Populus davidiana* Dode (Salicaceae) | 3 | 0.00 | 12.00 | 0.33 | 4.11 |
|  | *Ulmus pumila* Linn. (Ulmaceae) | 3 | 0.00 | 8.00 | 0.20 | 2.73 |

Supplementary Table 2. Spatial associations of the dominant tree species in both disturbed & undisturbed plots.

|  | Species | Scale (m) | | | | | | | | | | | | | | | | | | | | | | | | | | | | | | | |
| --- | --- | --- | --- | --- | --- | --- | --- | --- | --- | --- | --- | --- | --- | --- | --- | --- | --- | --- | --- | --- | --- | --- | --- | --- | --- | --- | --- | --- | --- | --- | --- | --- | --- |
|  |  | 0 | 1 | | 2 | | 3 | | 4 | | 5 | 6 | 7 | 8 | 9 | 10 | | 11 | | 12 | | 13 | | 14 | | 15 | | 16-20 | | | 21-25 | 26-30 | |
| Disturbed  plot | *Bp-Pk* | + | | + | | + | | + | | + | + | r | r | r | r | | r | | r | | r | | r | | r | | r | | r(+) | r(+) | | | r |
|  | *Bp-Pd* | r | | r | | r | | r | | r | r | r | r | + | + | | + | | + | | + | | + | | + | | + | | + | + | | | + |
|  | *Bp-Bc* | - | | - | | - | | - | | - | - | - | - | - | - | | - | | - | | - | | - | | - | | - | | - | - | | | -(r) |
|  | *Pk-Pd* | r | | r | | r | | r | | r | r | r | r | + | r | | r | | r | | r | | r | | + | | + | | +(r) | + | | | + |
|  | *Pk-Bc* | - | | - | | - | | - | | - | - | - | - | - | - | | - | | - | | - | | - | | - | | - | | - | -(r) | | | +(r) |
|  | *Pd-Bc* | - | | - | | - | | - | | - | - | - | - | - | - | | - | | - | | - | | r | | r | | r | | r | +(r) | | | + |
| Undisturbed plot | *Pk-Bp* | - | | - | | - | | - | | - | - | - | - | - | - | | - | | - | | - | | - | | r | | r | | r | r(+) | | | + |
|  | *Pk-Ta* | - | | r | | - | | - | | - | - | - | - | - | - | | r | | r | | r | | r | | r | | r | | r | r | | | r |
|  | *Pk-Am* | r | | r | | r | | r | | r | r | r | r | r | r | | r | | r | | r | | r | | r | | r | | r | r | | | r |
|  | *Bp-Ta* | + | | + | | + | | + | | + | + | + | r | + | + | | r | | r | | r | | r | | r | | r | | -(r) | - | | | - |
|  | *Bp-Am* | + | | + | | + | | + | | + | + | + | + | + | + | | + | | + | | + | | + | | + | | + | | r(+) | r | | | r |
|  | *Am-Ta* | - | | - | | - | | - | | r | r | r | r | r | r | | r | | + | | + | | r | | r | | r | | r | r | | | -(r) |

**Notes**: “+”: positive association, “-”: negative association, “r”: no spatial association; “+(r)”: more positive association points than no associations; “r(+)”: more no association points than positive associations; “-(r)”: more negative association points than no associations; “r(-)”: more no association points than negative associations.
